# 51. Sustained Impact of an Antimicrobial Stewardship (AS) Initiative Targeting Asymptomatic Bacteriuria and Pyuria in the Emergency Department (ED)

**DOI:** 10.1093/ofid/ofab466.253

**Published:** 2021-12-04

**Authors:** Mary Catherine Cash, Garrett Hile, James Johnson, Tyler Stone, Vera Luther, Vera Luther, Chris Ohl, James Beardsley

**Affiliations:** 1 Wake Forest Baptist Health, Winston-Salem, North Carolina; 2 Wake Forest Baptist Health System, Winston Salem, NC; 3 Wake Forest School of Medicine, Winston Salem, NC

## Abstract

**Background:**

The sustainability of unique AS initiatives are largely unstudied. A multi-faceted initiative to reduce inappropriate treatment of asymptomatic pyuria (ASP) and asymptomatic bacteriuria (ASB) in the ED was implemented at our institution in 2016. A pre-post intervention analysis demonstrated reduction in the inappropriate treatment (tx) of ASP/ASB from 100% to 32% (p< 0.001) following the intervention. The purpose of this present study was to determine the sustained impact of the initiative and determine if re-education provided in Oct 2020 could further reduce inappropriate tx.

**Methods:**

This was a retrospective, interrupted time series study conducted at an 885 bed academic medical center. Patients (pts) discharged from the ED in Nov 2019 – Feb 2020 (group 1) and Nov 2020 – Feb 2021 (group 2) were retrospectively screened in chronologic order until 50 pts in each group met study criteria. Similar to the 2016 study, pts were included if they were ≥ 18 years old and had a positive urine culture or pyuria. Pts were excluded if they had symptoms of a urinary tract infection (UTI), another infection requiring antibiotics (ABX), indwelling catheter, ureteral stent, or nephrostomy tube, or if pregnant or immunocompromised. The primary outcome was the proportion of pts prescribed ABX within 72 hrs of ED discharge. The secondary outcome was the number of pts returning to the ED with symptomatic UTI within 30 days of discharge. Group 1 was compared to the 2016 study’s post group to determine the sustained impact of the initial initiative; group 2 was compared to group 1 to determine the impact of re-education, which involved a presentation to ED providers and a posted algorithm and fact sheet.

**Results:**

Results from all time periods are summarized in Table 1. Improvement in inappropriate tx was still noted 3 years after the intervention (28% vs 32%; p = NS). Re-education did not further improve inappropriate prescribing, with 28% of group 2 pts still receiving tx.

Table 1.

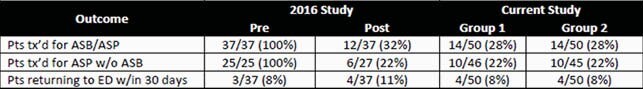

**Conclusion:**

The decrease in inappropriate use of ABX for ASP/ASB was still noted 3 years after implementation of a multi-faceted AS initiative. Re-education did not result in further improvement.

**Disclosures:**

**James Johnson, PharmD**, **FLGT** (Shareholder) **Vera Luther, MD**, Nothing to disclose

